# Learning-guided automatic three dimensional synapse quantification for *drosophila* neurons

**DOI:** 10.1186/s12859-015-0616-y

**Published:** 2015-05-28

**Authors:** Jonathan Sanders, Anil Singh, Gabriella Sterne, Bing Ye, Jie Zhou

**Affiliations:** 1 Department of Computer Science, Northern Illinois University, DeKalb, IL 60115 USA; 2 Life Sciences Institute and Department of Cell and Developmental Biology University of Michigan, Ann Arbor, MI 48109 USA

**Keywords:** Synapse detection, Automatic quantification, 3D confocal image, Model selection, BIOCAT

## Abstract

**Background:**

The subcellular distribution of synapses is fundamentally important for the assembly, function, and plasticity of the nervous system. Automated and effective quantification tools are a prerequisite to large-scale studies of the molecular mechanisms of subcellular synapse distribution. Common practices for synapse quantification in neuroscience labs remain largely manual or semi-manual. This is mainly due to computational challenges in automatic quantification of synapses, including large volume, high dimensions and staining artifacts. In the case of confocal imaging, optical limit and xy-z resolution disparity also require special considerations to achieve the necessary robustness.

**Results:**

A novel algorithm is presented in the paper for learning-guided automatic recognition and quantification of synaptic markers in 3D confocal images. The method developed a discriminative model based on 3D feature descriptors that detected the centers of synaptic markers. It made use of adaptive thresholding and multi-channel co-localization to improve the robustness. The detected markers then guided the splitting of synapse clumps, which further improved the precision and recall of the detected synapses. Algorithms were tested on lobula plate tangential cells (LPTCs) in the brain of *Drosophila melanogaster*, for GABAergic synaptic markers on axon terminals as well as dendrites.

**Conclusions:**

The presented method was able to overcome the staining artifacts and the fuzzy boundaries of synapse clumps in 3D confocal image, and automatically quantify synaptic markers in a complex neuron such as LPTC. Comparison with some existing tools used in automatic 3D synapse quantification also proved the effectiveness of the proposed method.

## Background

The subcellular distribution of synapses is fundamentally important for the assembly, function, and plasticity of the nervous system, and disruption of synapse development has been implicated in many types of neurological disorders [1–9].

Recent advances in imaging technologies now allow us to go beyond the traditional measure of synapse formation and dendritic spines, and to study the distribution of the synapses themselves in a three-dimensional context. Automated and effective quantification tools are a prerequisite to large-scale studies of the molecular mechanisms of subcellular synapse distribution. Current analysis is largely manual or semi-manual and often limited to arbitrarily selected parts of the dendritic field. Such practices make the process labor intensive and sometimes subjective.

However, computational challenges exist for the analysis of new data due to several reasons. The first issue is the large size of higher-dimensional biological image sets, which greatly increases the computational demand on the model and renders the design of an efficient and effective algorithm difficult. In addition, noise, staining artifacts and the optical limit (light microscopy can cause fuzzy images due to wavelength limit) prevent effective and precise detection; the anisotropic nature of the confocal images (xy resolution is higher than the z direction) requires special algorithm design. Moreover, accurate quantification is made challenging when crowded objects form clumps with large morphology or images display high contrast variation from region to region. Therefore, automatic synapse quantification from large, multichannel, high-dimensional confocal images requires special consideration to achieve the necessary robustness and efficiency.

While automatic quantification tools are available for 2D image analysis [10–12] and there are studies for quantifying other 3D biological objects than synapses (e.g., cells/nuclei) [13–16], existing tools for 3D synapse quantification are not yet sufficient. For example, automatic synapse detection based on connected component analysis falls short in quantifying clumps of multiple synapses. Threshold-based approach or variants compromise robustness for applicability to large high-dimensional images [5, 17]. On the other hand, sophisticated segmentation methods, such as those based on level sets, are less efficient and may have issue extending to 3D due to the resolution disparity between xy-z directions in confocal imaging. They also often involve human interaction and are thus not suitable for large, high-dimensional images on a regular PC.

To overcome these problems, we utilize an automatic learning-based method for effective and efficient quantification. Central to our method is a multidimensional discriminative model learned from reliable 3D feature descriptors*.* When discriminative models have been found effective in automatic 2D image recognition tasks [11, 18–20], the general consensus on 3D biological images has been that a discriminative model can also lead to more robust quantification results with 3D images [15, 21, 22] and is suitable for large-scale analysis, due to minimal user intervention once the model is trained, which is a good property for large-scale data analysis, as is necessary in genetic screening [23]. However, the application of discriminative models to 3D biological images has lagged behind their successful 2D counterparts. Other than the fact that the availability of large-volume 3D images is relatively recent, it may also be related to the need for 3D training sets and the lack of an ergonomic tagging tool using the 3D-WYSIWYG (What You See Is What You Get) strategy. The recent availability of the visualization tools such as Vaa3D [24], which allows for ergonomic tagging, aligned with the strong demand for automatic 3D quantification.

In this paper, we present a learning-guided approach for automatic 3D synapse quantification. We use a discriminative model to detect the synapses. The model output then guides automatic contour-based splitting to further improve the robustness of synapse quantification. Assisted by other modules such as multichannel co-localization and proximity analysis that will overcome staining artifacts, the process provides effective synapse-quantification for multichannel, high-dimensional light images. As the test system, we will use the lobula plate tangential cells (LPTCs) in the brain of *Drosophila melanogaster*, a system in which the subcellular localizations of gamma aminobutyric acid (GABA)-ergic synapses can be imaged in three dimensions using high-resolution confocal microscopy. In the Methods section, algorithms used to detect and quantify the GABA synaptic markers in LPTC’s axon as well as dendritic areas are described. The qualitative and quantitative results as well as discussions are then presented, followed by the conclusions.

## Methods

### Imaging synapses in single *Drosophila* LPTCs

The lobula plate tangential cells (LPTCs) in the brain of the fruit fly *Drosophila melanogaster* offer an in vivo system that allows for genetic manipulation and high-resolution imaging of subcellular localizations of GABAergic synapses [25–27]. These cells respond to directional movement of the visual field and are located in the optic lobe of the adult fly [28].

**Fig. 1:**
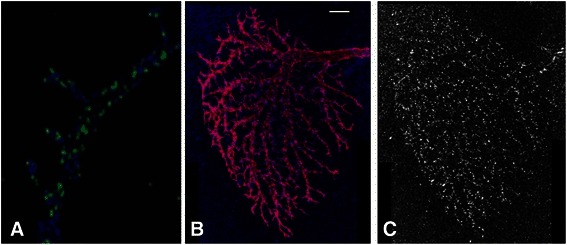
Raw images of the general morphology and GABAergic synapses a LPTC Horizontal System (HS) neuron. **a** The maximum intensity projection of the axon terminal. The blue channel is the axon morphology and the green channel is the HA-tagged GABA receptor RDL (RDL-HA). **b** The MIP view of the dendritic tree. The red channel is the tree morphology and the blue channel is the GABA receptor marker RDL-HA. Scale bar: 10 μm. **c** RDL-HA in the dendritic tree

Figure 1 shows maximal intensity Z-axis projections of 1024×1024×19 pixel laser-scanning confocal (LSC) images of a LTPC neuron. Using mosaic analysis with a repressible cell marker (MARCM) ([29], we visualized at single neuron-resolution the distribution of the postsynaptic GABA receptors labeled by a hemagglutinin (HA)-tagged GABAergic receptor subunit RDL (RDL-HA) [30] and the overall cell morphology marked by mCD8-monomeric RFP (mCD8-RFP) [31]. Figure 1a shows the axonal terminal of the LPTC neuron with GABAergic synapses labeled by RDL-HA. Figure 1b and c shows the dendritic arbor of a LPTC. The fluorophores used to label RDL-HA and mCD8-RFP were Cy5 and Rhodamine Red-X, respectively. For inhibitory synapses labeled by RDL-HA, the excitation was 633 nm and the emission was 670 nm (Cy5). For overall morphology labeled by mCD8-RFP, the excitation was 543 nm and the emission peak was 590 nm (Rhodamine Red-X). These fluorophores were scanned separately using sequential scanning.

The stained samples were imaged on a Leica SP5 LSC system with a 63x oil-immersion lens (numerical aperture = 1.40) in conjunction with Leica acquisition software. A digital zoom of 3 was applied. The pixel size was 80 (x) x 80 (y) x 400 (z) nm. Six frame averages and 4 line averages were applied to reduce random noise occurred during imaging acquisition.

Images were then deconvolved with the Huygens software. Theoretical point spread function (PSF) was used for deconvolution. The “signal-to-noise ratio” parameter, which is a noise filter in the Huygens software, was set at 20. After deconvolution, the 3D images of separate parts of a neuron were stitched together manually with the assistance of the Amira software.

### Overall algorithm design for 3D synapse quantification

**Fig. 2:**
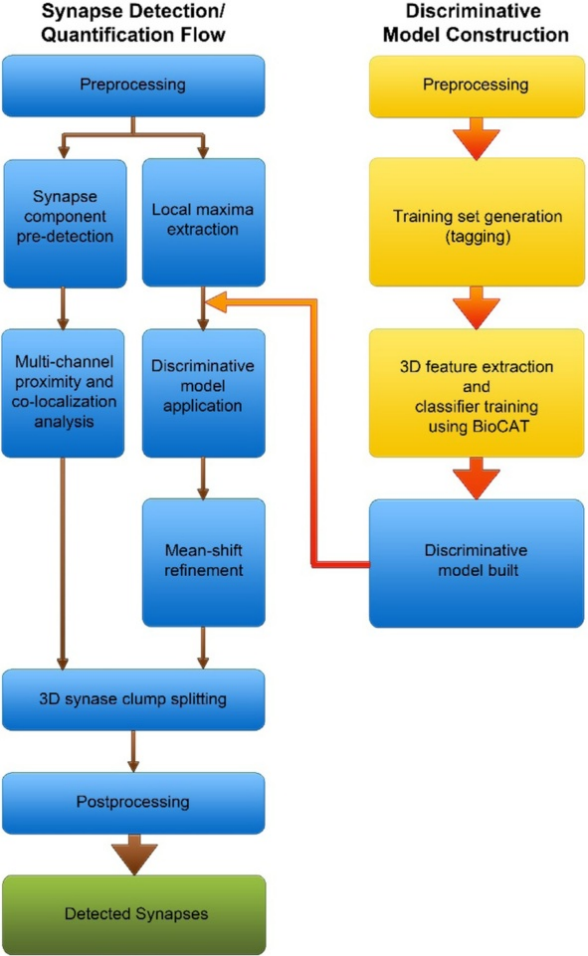
Flow chart of the 3D synapse quantification algorithm

Figure 2 illustrates the overall design of our automatic method for 3D synapse detection. The two-channel 3D image of synapses on LPTC axon or dendrite was first split into two images corresponding to the synapse channel and the morphology channel. The learning-based synapse detection algorithm was applied to the channel of GABAergic synaptic markers. A 3D discriminative model was constructed to detect the center of a synapse based on 3D features. One important step in the process is to select an optimal model for synapse detection, which utilizes a tool we developed called BIOCAT for BIOimage Classification and Annotation Tool (http://faculty.cs.niu.edu/~zhou/tool/biocat/). BIOCAT is a platform for biological image classification. In this project, it was used to compare the algorithm chains of 3D feature extraction/selection and classification algorithms in order to determine what algorithms were best suited to detecting centers of synaptic markers. Once the synapses were detected, the morphology channel was merged back for the purpose of co-localization analysis to filter out false positives based on the spatial proximity of synaptic markers and the axon/dendrite morphology. Synapse clump splitting was then performed guided by the predicted synaptic centers to yield the final quantification results. The components will be described in detail in next several sub-sections.

### Training and chain selection for synapse learning

A tool was created from our work on 2D bioimage annotation using a discriminative model, which we extended to higher dimensional images and named BIOCAT [32, 33]. BIOCAT has gone beyond image annotation to provide a flexible and extensible platform for biological image learning based on discriminative models, handling both image sets and the regions of interest (ROI) in images. A user-friendly graphical interface is included. State-of-the-art algorithms from pattern recognition and machine learning are included as plug-in modules. The modules include feature extractors such as scale-invariant feature transform [34], wavelet transform [35], Hessian and structural features [36]. Candidate modules also include classifiers such as a support-vector machine [37] and adaptive boosting [38]. The extensible design of BIOCAT has made it easy to add new algorithms as plug-ins. In addition, BIOCAT has an increasing focus on developing multi-dimensional descriptors that are large scale aware. Such descriptors can be especially useful for learning tasks involving high dimensional and high volume bioimages that require special attention to algorithm efficiency. It is an ongoing effort to develop novel and suitable algorithms. See BIOCAT website for latest updates.

The functionality of BIOCAT that is most pertinent to the learning-based synapse detection is the selection of an effective discriminative model. Currently, the common practice for model selection in pattern recognition and learning community is manual, trial-and-error comparison, largely based on the practitioner’s level of experience. BIOCAT, on the other hand, enables automatic model selection for biological-image classification and detection, and thus provides an adaptive tool for building models best suited to the quantification task.

The problem of synapse detection was formulated as the binary classification task of detecting the centers of synaptic markers. The discriminative model was trained based on a set of voxels, which were either the centers of synapses or not, to form a binary predictor. 3D ROIs in the training set were sized 9*9*3 pixels, surrounding a voxel that was either a synaptic center (positive) or not (negative). 25 positive ROIs and 25 negative ROIs were randomly chosen for learning purposes. In contrast to the large amount of synapses in the image, e.g. thousands of GABAergic synapses in a LPTC dendrite tree, the size of the training ROIs was rather small and easy to collect and tag. We did the tagging using ImageJ in a slice by slice fashion and then visually validated in the 3D volume view using the Vaa3D tool [24].

**Fig. 3:**
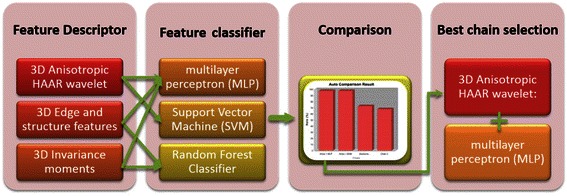
Algorithm chain selection for 3D synapse detection using BIOCAT

The ROI training set was then loaded into BIOCAT for chain selection. In BIOCAT, a discriminative model is represented as an *algorithm chain* which is defined as a sequence of several pattern recognition algorithms that include some feature extractors, one or more optional feature selector(s), and a classifier, which can also be an ensemble of multiple classifiers [33]. Examples of algorithm chains are demonstrated by Fig. 3. The algorithm chains were built and compared by BIOCAT. We used five-fold cross-validation to yield the recognition accuracy as the measure for comparison.

For the purpose of 3D synapse detection, several feature descriptors and their combinations were used to build the first components for candidate BIOCAT algorithm chains. Next, several variations of feature classifiers were paired with feature descriptors to complete the candidate chains. The chains involved in our experiments were derived from the components described below.

Candidate 3D feature descriptors extracted from its surrounding 3D volume for a voxel were determined using:

- 3D Anisotropic HAAR wavelet: A Multi-scale wavelet features extended to 3D and adapted to the anisotropic nature of 3D confocal imaging. It was defined in [33].
- 3D Edge and structure features [39].
- 3D Invariance moments [40].

Features were classified using the following candidate classifiers:

- Learning using neural network: a multilayer perceptron (MLP) with windowed momentum. The windowed momentum is added to improve the performance [41].
- Support Vector Machine (SVM) [37] .
- Random Forest Classifier [42].

Please note that BIOCAT provides more algorithm modules than those are described above. Those described here were more extensively tested with different parameter settings during the building and comparing of algorithm chains for 3D synapse detection.

**Fig. 4:**
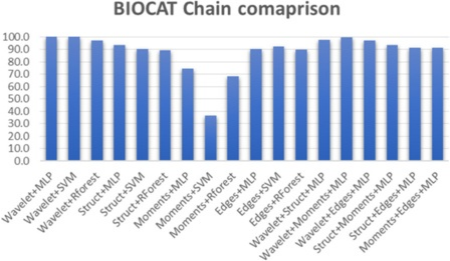
Comparison of 18 algorithm chains for 3D synapse learning

Figure 4 lists the results of comparing 18 algorithm chains that include the above features and classifiers as well as their various combinations.

The most powerful feature descriptor for determining synaptic markers based on 5-fold cross-validation was experimentally determined to be 3D anisotropic wavelet extractor module. Anisotropic HAAR wavelet is an efficient feature extractor that adapts to the anisotropic nature of 3D confocal imaging. It extracts multi-scale HAAR features from x-y planes and then sums up the features of all z slices with middle z slices weighted heavier than other slices. The anisotropic features logically complement the z-directional resolution disparity of confocal images and avoid the expensive full 3D extension of wavelet features.

The highest performing chains are the wavelet features combined with the Multi-Layer Perceptron module (MLP) or the Support Vector Machin (SVM), both achieving a 100 % cross-validation rate on the training set. MLP, an artificial neural network based learning algorithm, is a powerful classifier that has shown in other case of 3D object learning from microscopic images [43]. Experimentally, we found that a windowed momentum increased speed and also lead to slightly better performance than the traditional MLP (results not shown). We can notice from Fig. 4 that the choice of classifier is not as critical as the descriptors. 3D anisotropic wavelet features performs reasonably well with a variety of classifiers. For example, a chain with 3D anisotropic wavelet features and random forest classifier also yielded reasonable results in training set cross-validation. MLP was chosen as the classifier for our purpose due to its high accuracy and known faster testing time than SVM. On the other hand, some other feature descriptors, such as 3D Edges features or 3D moment features, yielded poorer than expected results. Conclusively, the learning algorithms of 3D anisotropic wavelet extractor combined with a Multi-Layer Perceptron with a windowed momentum is chosen as the learning algorithm for 3D synapse detection in this paper.

Since the training ROIs were of small size and the set size was also small, the training time for the chain was around 5 seconds on a regular PC. This is the time for learning the model. Once a model is learned, the time to make a prediction (for synapse detection) is very fast.

### Testing process and synapse detection

The learned model was then applied to the synaptic channel of the microscopic image to detect synapses. Specifically, the model was applied to the surrounding ROI, sized same as the training ROIs, of the remaining image voxels. The model then yielded a prediction whether or not that voxel was a candidate of a synaptic center. Applying the model on every single voxel, however, would not be an efficient choice for large 3D images in terms of time. Considering that the majority of voxels in the synapse channel were backgrounds for large 3D volumetric images, speed can be improved by obtaining the local maxima first and applying the discriminative model to those voxels only.

To obtain the local maximum voxels, a mask of foreground pixels was obtained and then a morphological filtering was conducted on the mask. The mask of foreground pixels as a base of extracting local maxima was typically obtained by a histogram-based thresholding [44]. But that method turned out to be insufficient for our purpose. It was due to the large size of the image and the variations among different local regions. We instead used Robust Adaptive Threshold Selection (RATS) [45].

RATS is a local adaptive thresholding method that uses estimates of the image gradient to determine local threshold values. To achieve robust thresholding, RATS divides the image into a quadtree hierarchy of sub-regions. The smallest region is called the leaflet. RATS uses the gradient weighted sum of the pixels to determine the local leaflet threshold [45]: $T = \frac{{\displaystyle \sum}\left({G}_{x,y}^2*{I}_{x,y}\right)}{{\displaystyle \sum}\left({G}_{x,y}^2\right)}$ , where G_x,y_ are the derivative approximation from a Sobel operator, and I_x,y_ is the intensity of the pixel. If the sum of the gradients didn’t exceed a minimum noise input parameter, the regional threshold of the parent region was used. This step was repeated recursively. The thresholds were then bilinearly interpolated across the regions to get a binary mask. The advantage of RATS is its local adaptability on microscopic images with contrast variations.

Full extension of RATS to 3D will involve interpolation of all the neighboring voxels in 3D. It will be computationally expensive for an image with a big size such as the dendrites of an LPTC neuron. Since most synapses are a few pixels deep in our confocal images, we used a pseudo 3D RATS for our purpose. We applied the RATS to the image stack slice by slice, based on the same parameter that was set using a reference slice. The z-slices close to the boundary are typically darker so the middle slice was used as the reference slice to avoid over-detection on other slices.

RATS may also be used as a segmentation method to detect foreground structures in images, which we will make use in the section on multi-channel validation. When providing a base for local maximum detection, we needed a mask so that all the local maxima were included. The parameter of RATS were set to obtain such an overmask. A 3D morphological filtering was then applied on the RATS mask. 3D search locality of 7*7*5 of the filter was used in obtaining the local maxima. Once the local maximal voxels are detected, the ROI of size 9*9*3 around each local maximal voxel was extracted, and passed to the discriminative model trained on the sample ROIs. For each positively detected synapse center, an iterative mean-shift procedure was performed to refine the location to the nearby center of mass. Two centers were then merged if they were closer than a fraction of expected synapse size after shifting. The procedure yielded the positively identified synaptic centers by the model.

### Multi-channel validation and synapse clump splitting

To improve the robustness of synapse detection, two more steps were employed: synapse clumps that contain more than one synapse center were split and the morphology channel of the dendrite or axon images was used to verify the synaptic marker. The multi-channel validation was simply based on the proximity of the detected centers and the foreground dendrite or axon morphology: Those detected centers that were not in the spatial proximity of 9*9*5 of the axon/dendrite structure were discarded as staining artifacts. So we focus on the algorithm of synapse clump splitting in this section.

The splitting of the clump was guided by the discriminative model from the previous prediction of the learning model. The split was done such that the detected synaptic centers fell into individual synapses, and at the same time optimally divide the clump based on contour and shape. This step was to obtain size quantification and refine the locations of synapse centers.

3D connected component analysis based on 26 neighborhood connectivity [46] was performed after RATS segmentation to get the object shape. Objects of very small sizes (less than 8 voxels) were discarded as noise. Concave contour points were first detected based on the maximum intensity projection of the synaptic objects obtained via RATS segmentation, and the line connecting an optimally selected pair of concave contour points will be used to segment the clump.

For each split, an optimal pair of two contour points {i, j} is chosen to segment the synapse by maximizing the functional, provided that the pair can separate the component to two parts so that the centers are on different sides:

1 $$ E\left(\left\{i,j\right\}\right) = \frac{C(i)*C(j)}{d\left(i,j\right)} $$

where *c(i)* is the concave score for the contour point *i* [47, 48], *d* is the Euclidean distance between the two contour points *i* and *j*.

The goal of Eq (1) was to find a pair of contour points that are highly concave and close to each other in Euclidean distance. In addition, the split put the detected markers on different sides. Concavity of a contour point *i* was calculated based on the following:

2 $$ \mathrm{C}(i) = {\displaystyle \sum_{k=i-1}^{i+1}}{w}_k $$

Where W_i_ is number of foreground pixels in the 5*5 window centered at point *i*. For more robustness, the two adjacent contour pixels (i-1) and (i + 1) were also involved in the concavity measure.

3D convex objects like imaged synapses can theoretically be segmented by determining the identity of a voxel using majority voting on three maximum-intensity projection planes. In anisotropic cases, the x-y plane often gives the most accurate result and suffices for our purpose of clump splitting.

For clumps involving more synapses, splitting were accomplished by consecutively separating one center from the rest until there is one center per synapse, as described in Table 1. One initial definition of Eq. (1) made use of the distances of two contour points along the contour to look for pairs of contour points for splitting. That approach worked for clumps with 2 centers. However, it tended to yield unnatural splitting in the case of big clumps with more than 2 centers that defied the oval-‘oid’ shape of biological objects. The guidance of the synaptic center markers during split was found to be a crucial condition for the goodness of the split.

**Table 1:** Algorithm for recursive splitting of synapse clumps

STEP 1	Perform maximum intensity projection of the detected 3D synapse object.
STEP 2	Obtain the contour points of the synapse cluster.
STEP 3	Calculate the concavity score of the contour points based on Eq. (2).
STEP 4	Choose a detected marker (denoted M1) from the markers pool P, whose sum distance from the rest of the markers is maximum.
STEP 5	Split M1 from the rest of the markers. Start from the highest concavity point, A, look for a point B so that segment AB splits marker M1 from the rest N-1 markers. If no such segment was found, move to the contour point with next highest concavity and repeat until a good split is found. When multiple segments AB were found, the optimal pair that maximizes Eq. (1) us used.
STEP 6	Assign new identity to voxels of the newly split synapse for quantification statistics.
STEP 7	REPEAT STEP 4, until P only has one marker left.

**Fig. 5:**
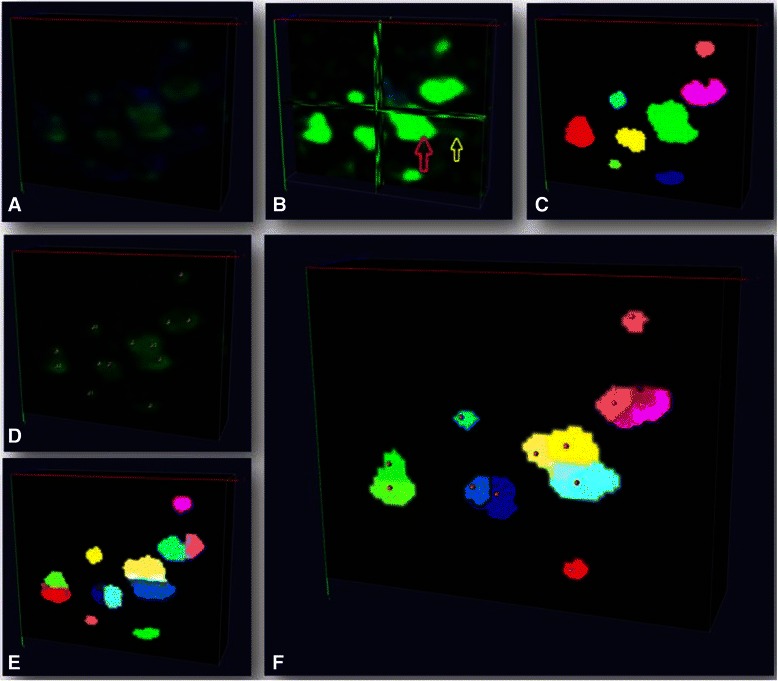
Synapse clump splitting. **a** Original image with green signal being GABAergic synapse. **b** Enhanced image with the yellow arrow indicating staining artifacts (dim green signal) and the red arrow indicates synapse clump. **c** synapse pre-detection (randomly color-coded). **d** Synapse centers detected using discriminative model; **e** model-guided splitting to split a clump into two synapses (randomly color-coded). **f** Example of synapses after iterative model-guided splitting (randomly color-coded). Visualization is done by Vaa3D

In Fig. 5, we exemplify the proposed approach for detecting and splitting synapses in a 3D confocal image that could not be resolved using intensity information alone. The centers of each detected and split synapse were considered as the refined synapse’s position to yield the final statistics of detected synaptic markers.

## Results and discussions

**Fig. 6:**
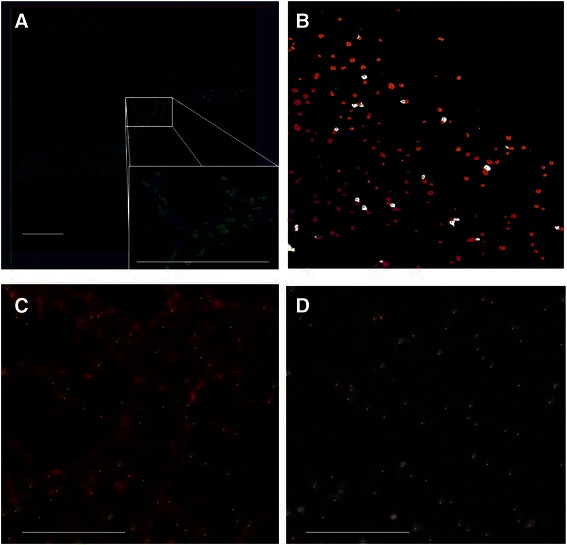
Examples of results of detected synapses for axon and dendrite. **a** A volume view of synapses on axon terminal. The blue channel is axon morphology, the green channel are GABAergic synapses. Red dots on the zoom-in view are the detected centers of synapes. Scale bar: 10 μm. **b** the detected synapses after model-guided splitting. The color coding is random. Shown is the maxiumn intensity projection of the 3D view of detected synapses. **c** Synapses detected on dendrites. Shown is a zoom-in region of primary branch. The red channel is the dendrite morphology by membrane staining. Green channel is GABAergic synapses. Red dots are the detected centers of synapses. Scale bar: 5 μm. **d** the same region of c but only the synaptic channel is shown. The dots are the detected centers of synapses. Scale bar: 5 μm. Visualization is done by Vaa3D a, c, d and ImageJ b

Figure 6 demonstrates the results of synapse detection on axon and dendrite images of LPTC, each contains hundreds to thousands of synaptic markers. Figure 6a) c) and d) show the detected centers overlaid on the original images. Visually we can see that our method is overall effective in detecting synapses. On Fig. 6d) we can see that the method is robust against staining artifacts that are present in the image. Figure 6b) exemplifies the result of detected synapses after splitting. We can see clumps of multiple synapses were split reasonably. Total numbers of synapses detected were 208 on the LPTC axon terminal image and 2932 on the dendrite image.

**Fig. 7:**
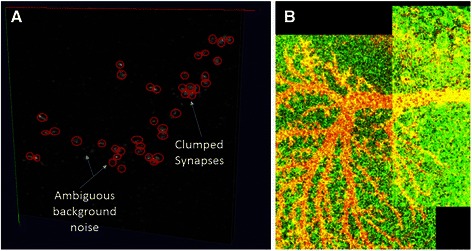
Illustration of manual annotation and stitching. **a** Manual annotation. Red circles exemplify the annotated markers in the presence of clumped synapses and ambiguous background noise. **b** Contrast-enhanced image to show the boundary of stitching. Visualization is done by Vaa3D

To further derive quantitative measure on the algorithm’s effectiveness, we manually annotated 5 regions in the LPTC axon image and 10 regions in the LPTC dendrite images. The regions were randomly chosen from the image, with the condition that the region contains reasonable amount of synapses (at least 10). The tool we used to annotate in the 3D volume image was Vaa3D [24]. Opinions from two computer scientists and two neuroscientists were collected during the annotation of the markers to reach a consensus (Fig. 7a gives an example of the manual annotation). These manual annotations in the synapse channel were used as references for deriving quantitative measures of precision and recall described below.

Precision and recall statistics of the 3D synapse detection algorithms were then calculated on each of these regions. It was done by performing a comparative analysis of the detected markers that fell within the boundaries of each manually annotated region of the whole image. First, we calculated the precision by counting the precisely detected synaptic markers that fell in the image region with respect to the total detected markers by the algorithm. A detected marker was defined as a precise one if it was within the 7*7*5 proximity of a reference synaptic center. Similarly, successfully recalled markers were defined as detected markers that are near the reference markers, within the same bounds as used for precision. Overall recall for a region was then calculated as the number of recalled markers divided by the total number of annotated markers in the region. These two measures are combined as their harmonic mean to yield an aggregate F-measure assessing the overall accuracy of the algorithm on each region with the F-measure = 2* (Precision * Recall)/(Precision + Recall). For a fair comparison with other object detection/segmentation methods, only the signal in the synapse channel was used for all methods when reporting the results.

Tables 2 and 3 list the precision, recall and F-measure on axon images and dendrite images, respectively. We can see from Table 2 that the synaptic center learning model delivered an effective result with a precision of 87.58 % and an F-measure of 84.98 % on axon. After the detected centers were further refined based on splitting guided using the detected markers, the results were further improved. The average F-measure was improved to 89.80 % and the average precision was improved to 93.27 %.

**Table 2:** Results of synapse detection on axon terminal using proposed method

Region	Precision	Recall	F-Measure	Precision	Recall	F-Measure
Method	Synapse Learning Model			After Model-guided Splitting		
Region 1	92.86	82.69	87.48	94.29	82.69	88.11
Region 2	94.12	84.62	89.11	97.62	81.54	88.86
Region 3	76.92	86.67	81.50	83.33	93.33	88.05
Region 4	90.67	82.80	86.55	95.45	87.10	91.08
Region 5	83.33	77.42	80.27	95.65	90.32	92.91
AVERAGE	87.58	82.84	84.98	93.27	87.00	89.80

**Table 3:** Results of synapse detection on dendrite using proposed method

Region	Precision	Recall	F-Measure	Precision	Recall	F-Measure
	Synapse Learning Model			After Model-guided Splitting		
Region 1	76.92	76.22	76.46	87.50	76.00	81.35
Region 2	83.33	41.67	55.56	83.33	50.00	62.50
Region 3	83.33	77.78	80.46	88.89	77.78	82.96
Region 4	84.21	83.33	83.77	89.47	83.33	86.29
Region 5	93.75	75.00	83.33	93.33	70.00	80.00
Region 6	92.31	57.14	70.59	92.31	57.14	70.59
Region 7	56.10	85.19	67.65	72.73	81.48	76.86
Region 8	95.65	88.00	91.67	95.00	76.00	84.44
Region 9	92.31	77.78	84.42	92.59	85.19	88.73
Region 10	62.96	82.35	71.36	56.25	88.24	68.70
AVERAGE	82.09	74.45	76.53	85.14	74.52	78.24

In Table 3, the synaptic center learning model delivered 82.09 % average precision. After model-guided splitting, the average precision was increased to 85.14 %. We see that most regions have good precisions but a few have low precisions. In addition to artifacts, image stitching could also be a source of error since the contrast disparity can be picked up by RATS. Figure 7b shows an example of such stitching. Working with stitched images more effectively can be a potentially important task in the future for large microscopic images from optical systems. The low recall regions were often due to the ambiguous artifacts related to intracellular transport.

### Comparison with other methods

We then compared our methods with other approaches that were used in synapse quantification, the 3D Object Counter [5, 46] and its variant. Considering that RATS has been used for generating mask in our process, we also compared to RATS when it is used as the segmentation method followed by 3D object counter to yield the count. The leaflet size of RATS was set such that it has a quadtree that is at least 5-level deep. For fair comparison, the RATS parameters were the same across all methods. For axon image, the leaflet size was set to 204; the minimum noise was set to 10, and the scaling factor was 3. For the dendrite image, they were set to 259, 4 and 3, respectively.

Table 4 lists the results of the original 3D object counter on the same regions that we have tested for the synapses on the axon terminal. Table 5 lists the results of RATS-variant of 3D object counter.

**Table 4:** Results for Synapse detection on Axon Terminal using the original 3D Object Counter

Region	Precision	Recall	F-Measure
Region 1	82.14	57.69	67.78
Region 2	93.10	60.00	72.97
Region 3	83.33	93.33	88.05
Region 4	90.24	55.91	69.05
Region 5	90.91	48.39	63.16
AVERAGE	87.94	63.06	72.20

**Table 5:** Result for Synapse detection on Axon Terminal using 3D Object Counter with RATS

Region	Precision	Recall	F-Measure
Region 1	92.00	63.46	75.11
Region 2	96.00	55.38	70.24
Region 3	81.82	86.67	84.17
Region 4	94.12	56.99	70.99
Region 5	90.00	45.16	60.14
AVERAGE	90.79	61.53	72.13

To compare with another learning-based tool, Table 6 lists the results with ilastik [49], which is an interactive learning and segmentation tool. Feature descriptors were chosen following the recommendation of the ilastik tutorial, which includes all features on a range of object sizes from 0.7px to the estimated size of target object. The comparison was performed on the synapses on axon because ilastik user interface is not responsive when working with the large dendrite image on a desktop with 8G RAM.

**Table 6:** Result for Synapse detection on Axon Terminal using ilastik

**Region**	**Precision**	**Recall**	**F**-**Measure**
Region 1	85.71	57.69	68.97
Region 2	88.24	63.08	73.56
Region 3	84.62	100.00	91.67
Region 4	89.13	62.37	73.38
Region 5	91.67	51.61	66.04
AVERAGE	87.87	66.95	74.72

Table 7 lists the comparison of summarizing precision rates, recall rates and F-measure of all the algorithms on the axon terminal. We can see that, while the traditional methods had an F-measure of around 72 %, our proposed method, with clump splitting guided by the synaptic markers detected from the discriminative model, gained the best F-measure of 89.80 %. Table 8 shows the comparison results on the dendrite which has a similar trend of increase.

**Table 7:** Comparison of results of synapse detection on axon

Algorithm	Precision	Recall	F-Measure
3D Object Counter	87.94	63.06	72.20
3D Object Counter + RATS	90.79	61.53	72.13
ilastik	87.87	66.95	74.72
Proposed: model output	87.58	82.84	84.98
Proposed: splitting guided by the model	93.27	87.00	89.80

**Table 8:** Comparison of results of synapse detection on dendrite

Algorithm	Precision	Recall	F-Measure
3D Object Counter	88.93	47.39	61.61
3D Object Counter + RATS	84.56	60.26	69.52
Proposed: model output	82.09	75.28	77.22
Proposed: splitting guided by the model	85.14	74.52	78.24

A statistical summary of size is also performed for all the detected synaptic markers between the size of 8 voxels and 1000 voxels. The maximum size of the detected object is 141 and 214 voxels for the axon and dendrite image, respectively. The minimum size is 8 voxels based as the set lower bound. And the average size of all the synaptic markers on the axon and dendrite images are 35.6 and 35.2 voxels respectively.

The results above were based on the synapse channel for a fair comparison. The morphology channel was not used and the colocalization analysis was not performed. Validation using the proximity analysis with the morphology channel reduced the number of synapses to 183 and 1984 for axon and dendrite. The removed ones were mostly staining artifacts or marker-like objects not associated with the neuron of interest.

## Discussion

From the results in Table 7 and Table 8, it can be shown that there was a marked increase in overall detection performance from the 3D object counter, to the learning model based marker detection, and finally the combination of segmentation and splitting with the learning model based detection results. The largest increase in model F-measure came from the model-based synapse detection. Using the results of model-based detection to supervise splitting was a logical extension to the algorithm flow and further increased accuracy.

This increase in effectiveness can be attributed to the algorithm robustly handling cases of false detections caused by noise and under-segmentation of adjoining synapses. Global threshold based object detection methods like Object Counter 3D cope poorly with background noise and variances in average intensity across large images. If the threshold criterion is tightened to attempt to eliminate noise, less intense foreground structures can be lost. It was also unable to handle clumps of synapses. Attempting to add more granular threshold approaches such as RATS reduces adverse effects from intensity variance, but is not enough to significantly improve results. The learning-based segmentation tool ilastik obtained better results than the thresholding-based approaches, but still fell short in overall F-measure. The results indicate that RATS or a simple pixel classifier by itself is not sufficient for robustly extracting the synaptic markers. In the proposed method, using a learning model based approach combined with adaptive thresholding allowed us to identify structures that are likely to be synapses. Model-guided synapse clump splitting enabled the method to further improve recall and precision.

Most segmentation methods start with detection of whole objects. Our learning model instead identified markers of possible synaptic centers. Our method thus naturally handled the cases of both isolated and adjoining synapses. The output of our supervised model for synapse center detection could then be combined with object detection. This pairing made up for the weaknesses of only object detection, and allowed for better guided splitting of clumps, even in denser regions. It led to the overall improved F-measure. This approach is expected to be suitable with confocal light microscopy where fuzzy clumps can be a common situation.

In addition to providing an effective way to work with fuzzy synaptic clumps, the benefit of our learning-guided approach is that the supervised learning model is easy to train and the model is stable and free of tuning after training. The BIOCAT tool also provides a flexible learning pipeline. The drawback is that it does involve extra work to annotate the training set, which is not always straightforward at first for non-technical users. For the case of synapse detection, we have found that a training set can be small (several dozen samples are often sufficient), so the needed effort for annotating the training set is also small, especially compared with the time-consuming and subjective effort of manually annotating hundreds or even thousands of synapses.

In future work, we will work on other types of synaptic markers to verify and improve the proposed process. For example, the synaptic markers imaged in the paper are post-synaptic receptors. We will incorporate other evidences such as the pre-synaptic markers to increase the reliability of synapse quantification. Manual annotation can also be improved for more comprehensive and accurate validation. We will also work on solving the contrast variations among stitched images duration quantification.

## Conclusion

In this paper, we presented a novel learning-guided synapse detection and quantification method for automatic recognition and quantification of synaptic marker using 3D confocal microscopic images of fruit fly neurons. The involved algorithms overcome the traditional methods’ shortcoming in handling cases of false detections caused by noise and under-segmentation of adjoining synapses, and were able to quantify a large number of synapses from the entire neuron.
